# Pediatric meningioma with a Novel *MAML2-YAP1* fusion variant: a case report and literature review

**DOI:** 10.1186/s12887-022-03747-8

**Published:** 2022-12-03

**Authors:** Xuan Zheng, Shaolei Guo, Dawei Liu, Jianping Chu, Yongfu Li, Xiaoxuan Wang, Xing Zhang, Chao Song, Quan Huang

**Affiliations:** 1 Department of Neurosurgery, The First Affiliated Hospital of Sun Yat-sen University, Guangzhou, China; 2 Department of Pathology, The First Affiliated Hospital of Sun Yat-sen University, Guangzhou, China; 3 Department of Radiology, The First Affiliated Hospital of Sun Yat-sen University, Guangzhou, China; 4 State Key Laboratory of Translational Medicine and Innovative Drug Development, Jiangsu Simcere Diagnostics Co., Ltd, Nanjing, China; 5 Department of Medicine, Nanjing Simcere Medical Laboratory Science Co., Ltd, Nanjing, China

**Keywords:** Pediatric meningioma, *YAP1*, *MAML2*, Gene fusion, Next-generation sequencing

## Abstract

**Background:**

Pediatric meningioma with *YAP1* fusion is a rare subset of meningiomas. Currently, there are lack of integrated clinical, radiological, and pathological features on this subset. Here, we reported a case of pediatric meningioma with a novel *MAML2-YAP1* fusion variant and reviewed the relevant literature.

**Case presentation:**

We presented a case of 12-year-old boy with meningioma adjacent to the superior sagittal sinus and falx. Simpson grade II gross total resection was performed after diagnosis. Pathologically, he was diagnosed as WHO grade I meningothelial meningioma with rhabdoid features. A next-generation sequencing-based gene panel was performed to determine the molecular features for potential treatment, and a novel *MAML2****-****YAP1* fusion break point was identified.

**Conclusion:**

Pediatric meningioma with the fusion of *YAP1* and *MAML2* genes is more likely to have pathological features of rhabdiod cells, which needs to be validated in large-scale studies for exploring better treatment under the integrated diagnosis.

## Background

Meningiomas are the most common primary intracranial tumors, representing 20-30% of central nervous system tumors [1]. Pediatric meningioma only accounts for less than 1% of all meningiomas [2], which may differ from adult meningioma in clinicopathologic and molecular patterns. According to European Association of Neuro-Oncology (EANO) guidelines, *YAP1* fusion can be an oncogenic driver for sporadic pediatric meningiomas [3]. As a principal regulatory target in the Hippo signaling pathway, *YAP1* is involved in a variety of human cancers [4]. *YAP1* fusion was identified as a potential oncogenic driver in meningiomas by strengthening the deregulation of the Hippo pathway [5]. Several *YAP1* fusion partners have been found in recent years, such as *YAP1-PYGO1*, *YAP1-FAM118B*, *YAP1-MAML2 *[5, 6]. However, there are lack of integrated clinical, radiological, and pathological features of pediatric meningioma with *MAML2-YAP1* fusion. Herein, we reported a case of pediatric meningioma with a novel *MAML2-YAP1* fusion variant and reviewed the currently available literature.

## Case presentation

**Fig. 1:**
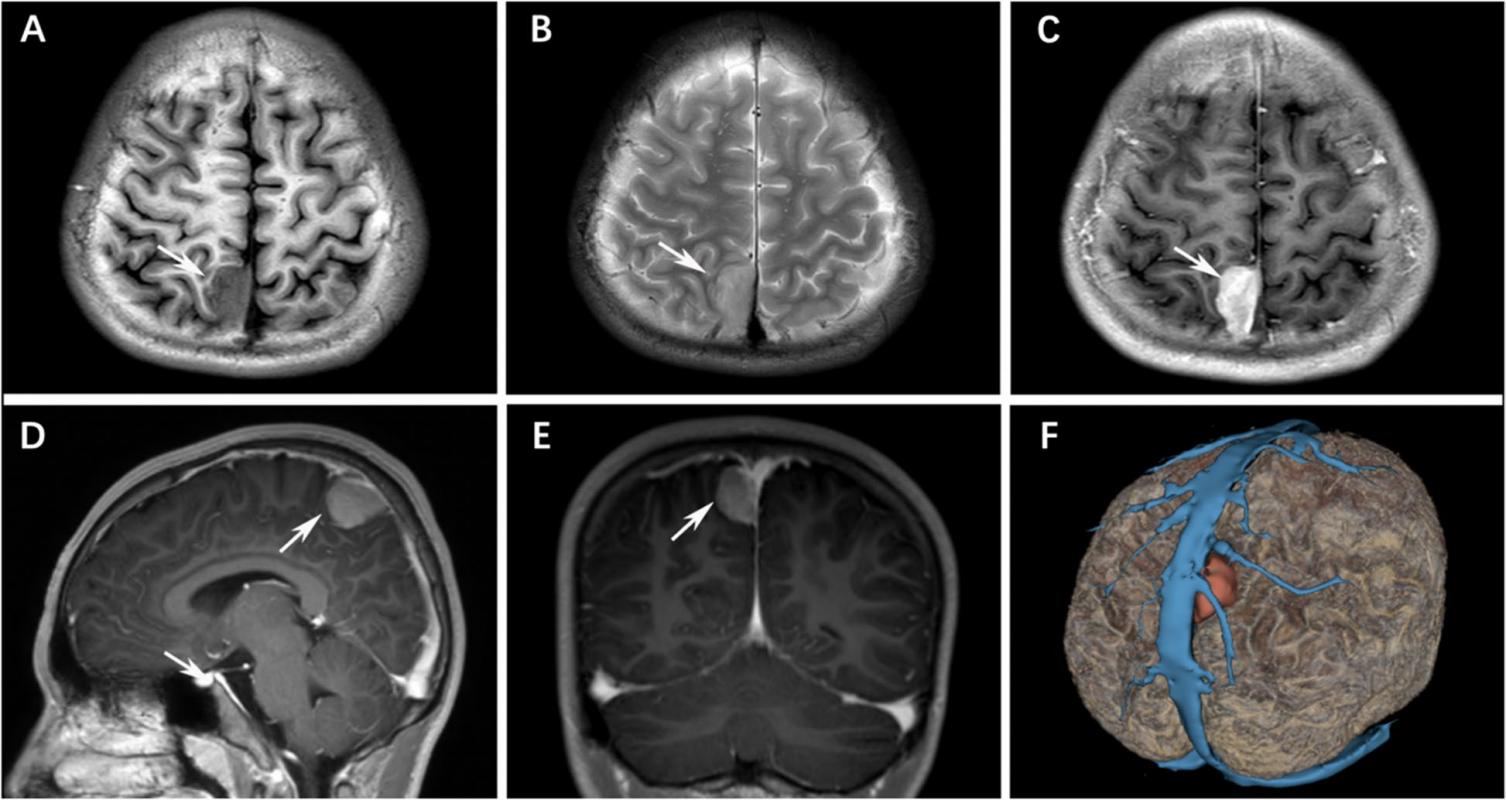
*YAP1-MAML2* fusion variant in a 12-year-old boy with meningioma adjacent to the superior sagittal sinus in the right parietal lobe. **A-C** Axial MRI shows a D-shaped, well-circumscribed and dura-based mass (arrow). The mass demonstrates isointense with cortex on the T1-weighted imaging **A** hyperintense on the T2WI sequence **B** and strong enhancement after the administration of Gd-based contrast agent **C**; **D-E** Sagittal and coronal T1-weighted MR imaging shows the mass enhances strongly after administration of Gd-based contrast agent, “dural tail” sign could be seen on both sagittal and coronal view. The tumor originates from the lateral wall of the sagittal sinus and the top of the cerebral falx. **G** Three-dimensional reconstruction is performed using Slicer (http://www.Slicer.org), which showed the upper Trolard vein not invaded by the tumor

A 12-year-old boy complained headache for 6 months. MRI revealed a D-shaped mass adjacent to the superior sagittal sinus and falx in the right parietal lobe. The mass was well-circumscribed and dura-based, with a size of 15 × 27 × 22 mm. No necrosis, cysts or hemorrhage was found. MRI also showed isointense with cortex on the T1-weighted imaging and hyperintense on the T2WI sequence, lightly hyperintense on the T2-Flair weighted imaging, but MRI enhanced homogeneously and intensely after intravenous administration of the contrast with gadolinium (Fig. 1). The gray matter next to the central gyrus was buckled. Dural tail sign was observed, suggesting a meningioma “en plaque”, but peritumoral edema was indistinct. Computerized tomography perfusion imaging further showed prolonged time to peak and mean transit time, as well as increased relative cerebral blood volume and flow.

**Fig. 2:**
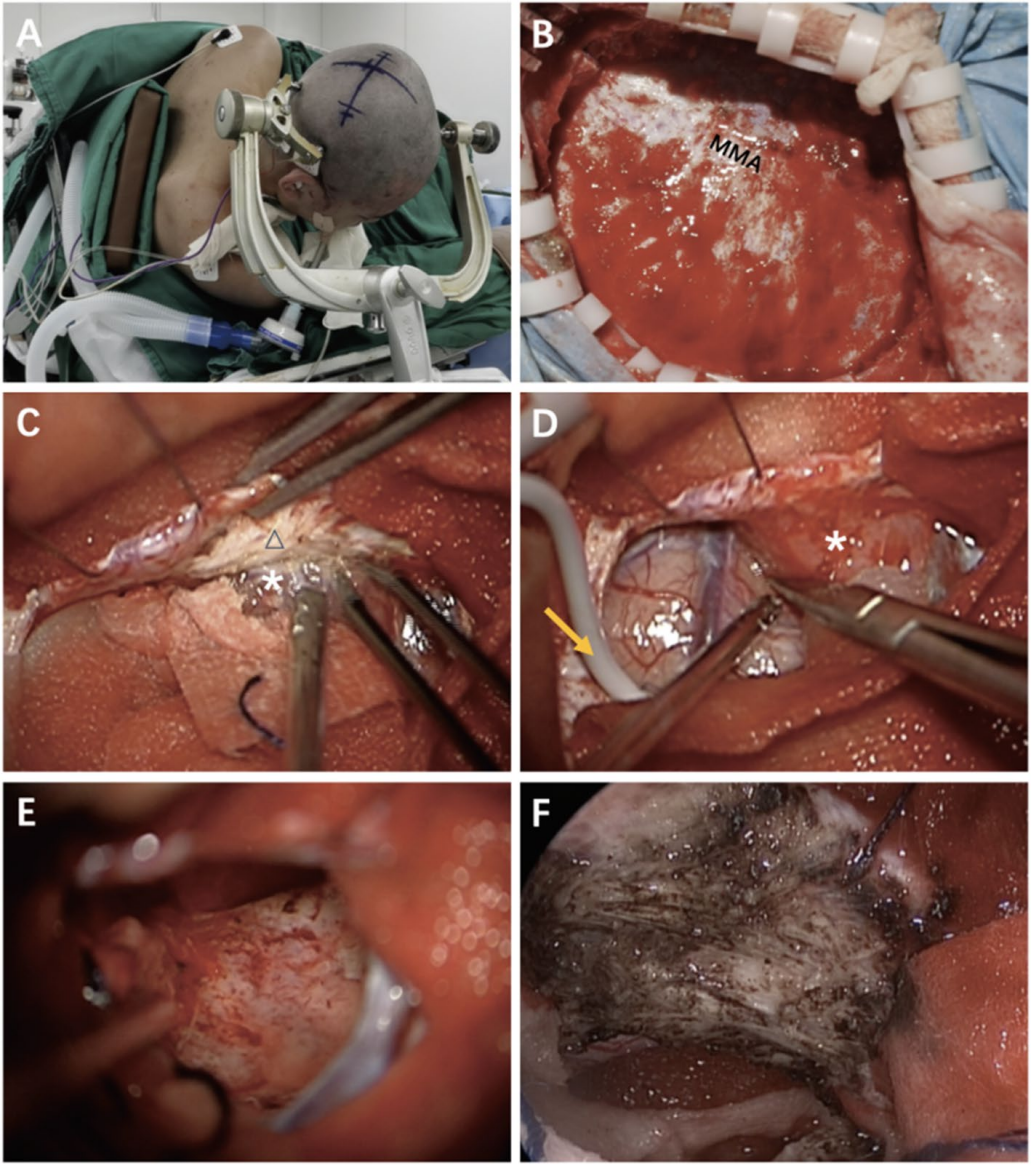
Surgical findings. **A** The patient in a right recumbent position; **B** After removing the bone flap during the craniotomy, we can notice that the dura mater is intact and not invaded; **C** The invasion can be seen in the right wall of the superior sagittal sinus; **D** The relationship between the tumor and the normal brain tissue is clear; **E** The normal brain tissue after complete resection of the tumor is intact but migratory; **F** The right wall of the sagittal sinus has been conducted by a bipolar electrocautery to achieve a Simpson grade II gross total resection. MMA: middle meningeal artery; △: sagittal sinus; *: tumor; arrow: electrocorticalgram

Simpson grade II gross total resection was performed in a right decubitus position. Through a craniotomy of 5.5 × 6.5 cm, we found his dural arteries and veins enlarged abnormally, while the bone flap had no signs of invasion (Fig. 2). The tumor was close to the centerline, its anterior edge adhered to the right vein of Trolard, and the right wall of the superior sagittal sinus and falx was invaded. The central sulcus in close to the tumor was located by the somatosensory evoked potential with cortical electrode on the cortex surface, and the epilepsy wave detected by the electroencephalogram was not found during the operation. The lobulated tumor with the size of 3 × 2.5 cm had a clear boundary with many nodules on the surface and adhered to the normal brain tissue, without full arachnoid membrane between the tumor and the brain. The strata externum of the sagittal sinus and falx was removed, and the inner of the sinus was kept intact. The bone flap was returned to the patient. No neurological adverse events occurred during the follow-up.

**Fig. 3:**
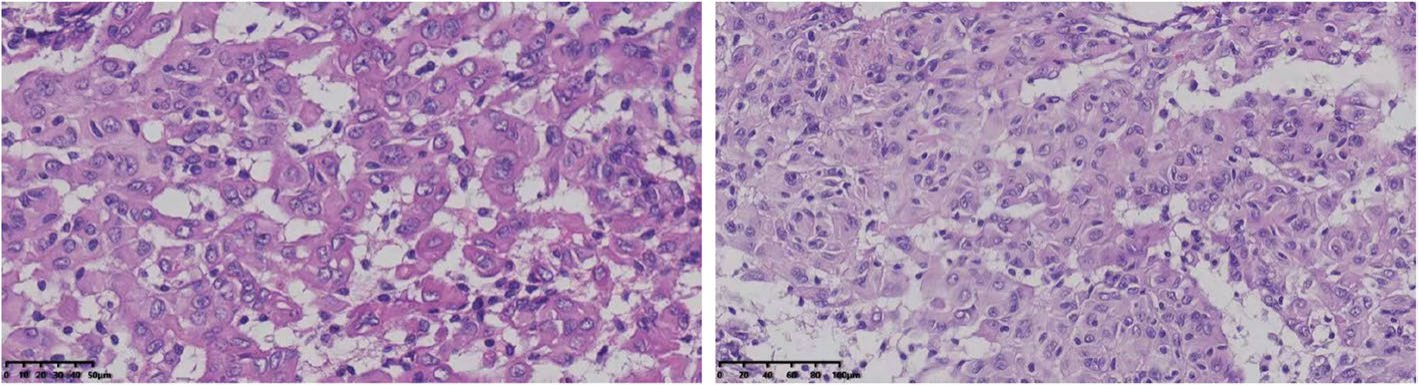
Histological images of rhabdoid cells

KF-PRO serial scanner was used, and pathological assessment was performed using K-Viewer software. Pathologically, the tumor cells manifested nested, sheet-like or whorled aggregates of spindle to epithelioid cells prominently with indistinct cell borders. Mitotic count was less than 1 per 10 high-power fields. Focally, rhabdoid cells were identified, accounting for 10% of the tumor (Fig. 3). Rhabdoid morphology was characterized by incomplete differentiation and intercellular adhesion, not accompanied by paranuclear inclusion body. Atypical features including brain invasion, hypercellularity, small cell formation, macronucleoli, sheeting architecture and spontaneous necrosis, were not identified in this tumor. Therefore, the patient was diagnosed as WHO grade I meningioma with focal rhabdoid features. The tumor cells showed diffuse and strong EMA and SSTR2 immunoreactivity. Immunohistochemistry for CD34, S100, STAT6, CK and SOX10 were negative in all tumor cells. Most tumor cells showed diffuse expression for SMARCB1/INI-1. The Ki-67 index was less than 1%.

**Fig. 4:**
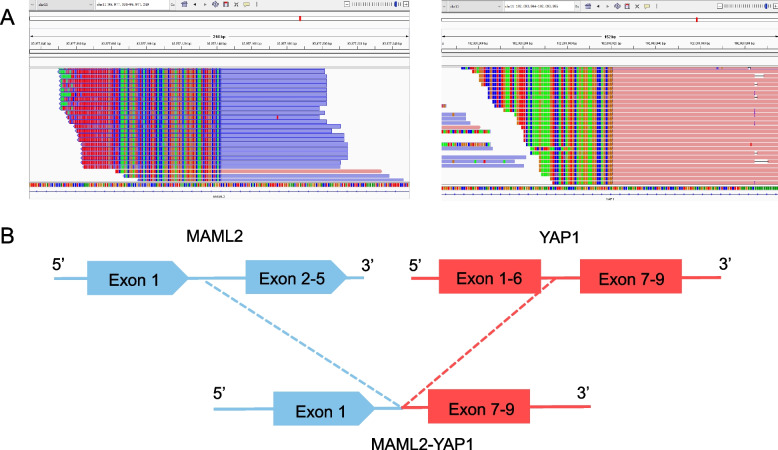
*YAP1-MAML2* gene fusion in a patient with meningoima. **A** Next-generation sequencing findings of the primary tumor tissue sample; **B** A novel *MAML2-YAP1* gene fusion break point

To determine molecular features and seek potential treatments, a next-generation sequencing-based gene panel (Simceredx, Nanjing, China) was used for genomic profiling in primary tumor tissue and matched blood. Except for a novel *MAML2-YAP1* fusion break point (5’ *MAML2* exon 1 fused to 3’ *YAP1* exons 7–9) identified (Fig. 4), no other mutations like single nucleotide polymorphism, InDel and copy number variations were detected.

## Discussion and conclusions

The fusion of *YAP1* and *MAML2* genes is mainly reported in low-grade pediatric meningioma. Previous studies had reported its different break points (Table 1), such as 5’ *YAP1* exon 1–5 fused to 3’ *MAML2* exons 2–5, 5’ *YAP1* exon 1 fused to 3’ *MAML2* exons 2–5, and more [5]. As a transcriptional co-activator, *YAP1* is the downstream effector of the Hippo pathway exerting effects primarily through TEAD family transcription factors and modulating the expression of genes involved in cell proliferation and apoptosis [7, 8]. Through *YAP1* overexpression, deregulation of the Hippo pathway is very common in human malignancies and has been considered a central mechanism in meningioma occurrence [5, 9]. There is a study suggesting that like other meningiomas, *YAP1*-fusion meningiomas have overexpression of EGFR and MET but are biologically distinct from *NF2*-driven meningiomas [6]. In this report, a novel *MAML2-YAP1* gene fusion break point was detected in a child with *NF2* wild-type meningioma, which may expand the genetic spectrum of somatic aberrations related to *NF2* wild-type meningiomas to involve the *MAML2-YAP1* fusion.

**Table 1:** The fusion break points of *YAP1* and *MAML2* genes in pediatric meningioma

Case #	Age	Sex	Tumor location	Gene fusion	Fused exons	WHO grade	Subtypes
1	4	F	Lateral ventricles, third ventricle	*YAP1:MAML2*	Ex5:Ex2	II*	Atypical*
2	1	M	Third ventricle, lateral ventricle	*YAP1:MAML2*	Ex1:Ex2	NA	NA
3	2	M	Skull base	*YAP1:MAML2*	Ex1 and 2nd intron:1st intron	NA	NA
4	17	M	Cavernous sinus	*YAP1:MAML2*	Ex5:Ex2	I*	Transitional
5	7	F	Parietal	*YAP1:MAML2*	Ex5:Ex2	NA	With focal rhabdoid features
6	7	F	Frontal	*YAP1:MAML2*	Ex5:Ex2	I	With rhabdoid features
7^△^	12	M	Parafalcine	*MAML2:YAP1*	Ex1:Ex7	I	With focal rhabdoid features

“*”: The initial diagnosis did not provide subtyping/grading and was added after reviewing the previous studies

“^△^” : represents the data from our case

NA: In some cases, there are no sufficient materials for additional histological workup

Currently, the pathological features of pediatric meningioma with the fusion of *YAP1* and *MAML2* genes remain unclear. This patient was diagnosed as a meningothelial meningioma with rhabdoid features. The “rhabdoid meningiomas” should have been rare WHO grade III tumors that tend to have an aggressive course, but this case showed no evidence of histological anaplastic or invasive features that a typical rhabdoid meningioma should have and had no other molecular alterations such as *TERT* and *CDKN2A/B*, thus WHO grade I was defined. Interestingly, another two cases of pediatric meningioma with *YAP1-MAML2* gene fusion in the previous study also showed rhabdoid features (Table 1). It may be assumed that pediatric meningioma with the fusion of *YAP1* and *MAML2* genes is more likely to have pathological features of rhabdoid cells.

In this report, we attempted to identify the potential genomic aberrations underlying the histological subtype of rhabdoid meningiomas. Characterization of such alterations is challenging due to uncommon anaplastic meningiomas, especially rhabdoid meningiomas, and significant interobserver variabilities in the diagnosis of this entity and in the recognition and description of rhabdoid features [10–12]. In addition, the rhabdoid subtype was initially defined as aggressive and exclusively high grade, but without significant high-grade histologic features, some meningiomas with rhabdoid cytomorphology showed indolent behaviors analogous to WHO grade I tumors [13], highlighting the genetic diversity of meningiomas with rhabdoid features. Currently, *BAP1* germline and somatic mutations have been identified to be associated with clinically aggressive meningiomas with rhabdoid features [12]. However, in our case, only *MAML2-YAP1* fusion was detected, but not *BAP1* germline and somatic mutations, which might be associated with infrequent rhabdoid meningiomas and interobserver variabilities. In the future, multi-institutional efforts are required to better characterize the clinicopathological and genomic features of meningiomas with rhabdoid features.

In our knowledge, research and development of drugs targeting *YAP1* may be a novel direction. Protein-protein interaction sites between YAP/TAZ and TEAD have been identified as a potential drug target of Hippo pathway [14]. One of the mechanisms is to directly inhibit α-helix or Ω-loop of the YAP-TEAD binding site, the other is to target the TEAD palmitoylation pocket to indirectly disrupt YAP/TAZ-TEAD complex and modulate Hippo pathway activity [15]. *YAP1* inhibitors have not entered the clinical stage in the field of oncology yet, but they might provide a new therapeutic direction in the future.

In conclusion, a novel *MAML2-YAP1* fusion break point in a child with meningioma was identified in our report, which expanded the *YAP1* fusion spectrum. This case not only provides integrated clinical, radiological, and pathological features of pediatric meningioma with the fusion of *YAP1* and *MAML2* genes, but also highlights the importance of integrated diagnosis in pediatric meningioma.

## Data Availability

The datasets generated and/or analysed during the current study are available in the NCBI Sequence Read Archive (SRA) repository (accession number: SRR22191684).
